# Force-Sensing Silicone Retractor for Attachment to Surgical Suction Pipes **†**

**DOI:** 10.3390/s16071133

**Published:** 2016-07-21

**Authors:** Tetsuyou Watanabe, Toshio Koyama, Takeshi Yoneyama, Mitsutoshi Nakada

**Affiliations:** 1 Institute of Science and Engineering, Kanazawa University, Kanazawa 9201192, Japan; toshio83@stu.kanazawa-u.ac.jp (T.K.); yoneyama@se.kanazawa-u.ac.jp (T.Y.); 2 Faculuty of Medicine, Kanazawa University, Kanazawa 9200934, Japan; mnakada@med.kanazawa-u.ac.jp

**Keywords:** force sensor, retraction, suction, force visualization mechanism

## Abstract

This paper presents a novel force-sensing silicone retractor that can be attached to a surgical suction pipe to improve the usability of the suction and retraction functions during neurosurgery. The retractor enables simultaneous utilization of three functions: suction, retraction, and retraction-force sensing. The retractor also reduces the number of tool changes and ensures safe retraction through visualization of the magnitude of the retraction force. The proposed force-sensing system is based on a force visualization mechanism through which the force is displayed in the form of motion of a colored pole. This enables surgeons to estimate the retraction force. When a fiberscope or camera is present, the retractor enables measurement of the retraction force with a resolution of 0.05 N. The retractor has advantages of being disposable, inexpensive, and easy to sterilize or disinfect. Finite element analysis and experiments demonstrate the validity of the proposed force-sensing system.

## 1. Introduction

A large number of medical devices are used in surgical operations. Examples of medical devices used for neurosurgery include the electric scalpel, forceps, retractor, bipolar forceps, endoscope, and suction pipe. Surgeons switch between these devices often, which leads to increased complexity of surgical procedures and longer operation time. However, these consequences are disadvantageous for patients. Thus, devices that are more functional are required; one way to achieve this is to combine multiple functions in a single device.

**Figure 1:**
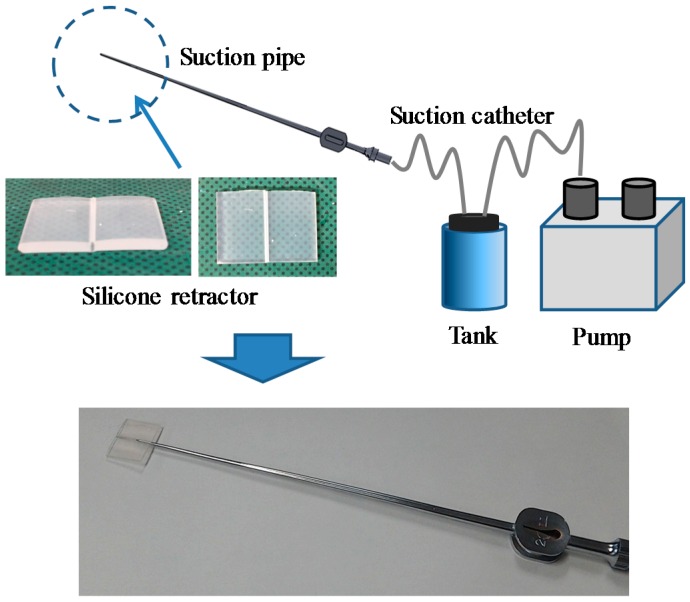
Suction pipe/device and silicone retractor for suction pipe.

This study focuses on suction pipes and retractors, which are the most frequently used devices in neurosurgery. Retractors are used to enhance the visibility of surgical spaces through pulling back of some part of the tissue. Suction pipes are used to remove blood and resection soft tissues. Given their fundamental functions, surgeons frequently switch between these devices and sometimes attempt to use the suction pipe as a retractor, although this is not always achieved successfully. To achieve better consistency, the neurosurgeons in the authors’ group developed a silicone retractor that can be attached to the tip of a suction pipe (see Figure 1). By extending the area to be retracted to the suction tip, surgeons can simultaneously retract and suction out tissues. The remaining issue is then used to measure the retraction force. The brain is the most critical human organ and, so, damage to brain tissues must be minimized. In addition, brain tissues are extremely soft and fragile. A force-sensing system is naturally expected to reduce the risk of damage to this organ. With this background, in this study, a force-sensing function was embedded in a silicone retractor attached to suction pipes. The main features of the resulting hybrid device are as follows:

**Force visualization**: The device employs a force visualization mechanism in order to aid surgeons in estimating the magnitude of the load applied on tissues. If a camera or endoscope is used, the force can be quantified at resolutions of up to 0.05 N over a range of 0–0.3 N.

**Easy setup**: Only silicone is used to construct the sensing part, and no electric components are present in the device. The sensing part is easily attachable to a suction pipe, and it has advantages of being disposable, inexpensive, and easy to sterilize or disinfect.

**Multiple functions**: The device enables simultaneous utilization of the suction, retraction, and retraction-force-sensing functions.

The present study is an extension of a previous study [1], containing additional results of an evaluation of the effectiveness of the proposed system when the target objects are curved elastic surfaces, similar to those encountered in real life. It should be noted that the curved elastic surfaces are rough and they are, therefore, the most difficult types of targets for the developed retractor. In our previous study [1], the system was evaluated only for target objects with solid and flat surfaces. The remainder of this paper is organized as follows. After a description of some related work in Section 2, the proposed force-sensing system is described in Section 3. The results of experimental evaluations are then presented in Section 4, and finally, the outcomes of the study are summarized in the concluding Section 5.

## 2. Related Work

A number of force and tactile sensors are available, some of which have been designed for use in medical surgery [2,3,4,5,6,7,8]. These sensing systems are targeted at providing haptic feedback to surgeons so that they can palpate and identify tumors in medical robotic systems. Strain gauges are commonly used in force-sensing systems [9,10,11], and electrical components are important for transferring the force information. However, medical devices require frequent disinfection or sterilization, which is difficult to do when they include electrical components. Additionally, components such as amplifiers are needed, making the overall system relatively large and expensive. Another problem is the noise in the wiring used for signal transfer. Other sensing approaches include the use of magnets in medical devices [12,13], but this results in the same issues of wiring noise and system size. The use of magnets also introduces the issue of MR compatibility. Consequently, force-sensing systems without electrical or magnetic components have been developed by several groups. For example, Takaki et al. [14] utilized moiré fringe patterns to embed a force-sensing function into forceps. Tadano and Kawashima [15] used a pneumatic servo system for the development of a force sensation feedback system, and Kawahara et al. [16] developed an organ stiffness measurement system by using an air jet and visual information from an endoscope. Peirs et al. [17] detected the deformation of flexible optical fibers embedded in forceps and estimated the force of the deformation. Tada et al. [18] constructed a force sensor based on the illumination change of a light source attached to an elastic frame. Puangmali et al. [19] presented a three-axis force sensor based on an optical sensing scheme. Polygerinos et al. [20,21,22,23] developed several types of force sensors for catheterization, which are also based on an optical sensing scheme. Liu et al. [24] proposed a wheeled probe for the identification of tissue abnormality. Ahmadi et al. [25] presented an MRI-compatible optical fiber tactile sensor that includes only a single moving part. Xie et al. [26] developed an optical tactile array probe head for tissue palpation. Tan et al. [27] developed an MRI-compatible three-degree-of-freedom (3-DOF) force sensor based on intensity modulation of optical fibers. Su and Fischer [28] developed an optical-fiber-based force/torque sensor for prostate needle placement. Su et al. [29,30] also developed an optical-fiber-based force sensor for needle insertion. Turkseven and Ueda [31] developed an optical fiber sensor for haptic feedback in robotic surgeries. Liu et al. [32] utilized low-coherence Fabry-Perot interferometry for developing an optical-fiber-based force sensor. Watanabe et al. [33,34] utilized a highly elastic fabric to visualize force information. They also developed a stiffness sensor based on visualization of force information [35]. However, none of these developed systems is capable of providing simultaneous retraction, suction, and force-sensing functions.

With regard to force-visualization-based sensors in other fields, Ohka et al. [36] detected a three-axis force from the poses of conical feelers, and Kamiyama et al. [37] estimated the force distribution from the poses of two-layer spherical distributed markers. Winstone et al. [38] developed TACTIP, a biologically-inspired vision-based tactile sensor. However, the required ranges of the sizes and forces of these developed systems are different from those of force-sensing systems used for neurosurgery. In particular, miniaturization is an important consideration for these force-sensing systems [6,7].

## 3. Force-Sensing System

### 3.1. Target Situation

**Figure 2:**
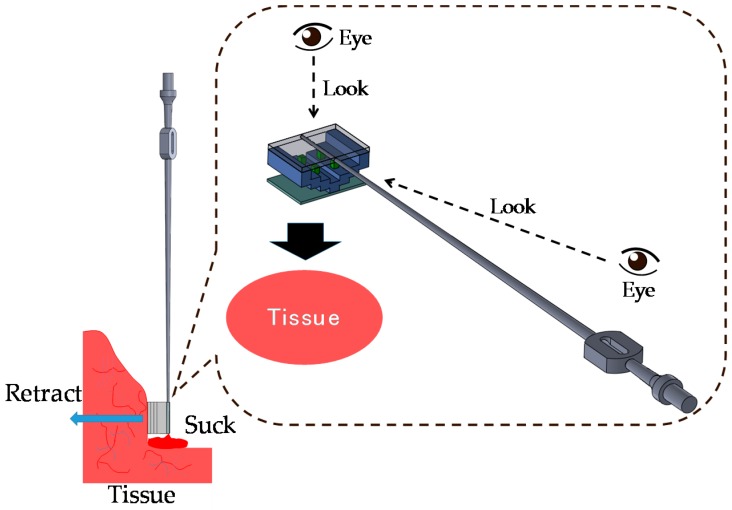
Target situation I: surgeon evaluates retraction force visually.

This study focuses on suction and retraction in neurosurgery. Figure 2 shows an example of a situation in which the developed silicone retractor is used. The retractor, which is embedded with a force-sensing function, is attached to the tip of the suction pipe. The suction pipe can be used to suck out blood, saline, and soft tumors to clear the visible space. The silicone retractor extends the visible area by retracting tissue. Using the force visualization function embedded in the silicone retractor, surgeons can estimate the magnitude of the retraction force and avoid fracture or breaking of normal tissues caused by application of an extremely large load.

**Figure 3:**
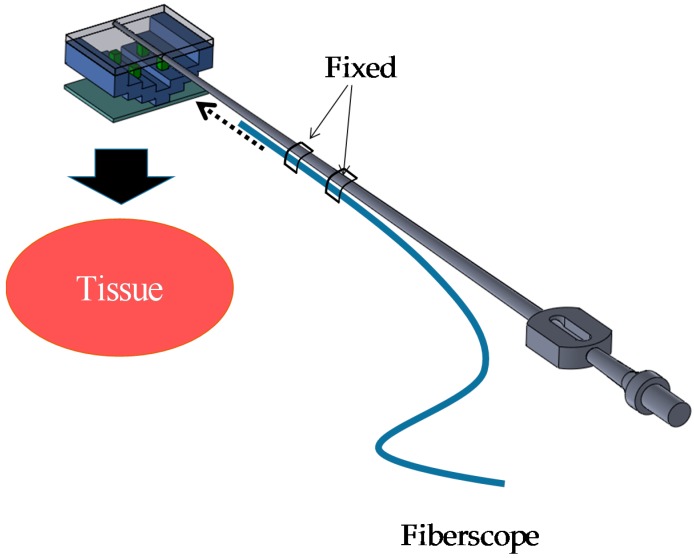
Target situation II: retraction force is captured with fiberscope.

In the situation shown in Figure 2, the visualized force information is slightly affected by the surgeon’s viewing angle. When accurate and quantitative force information is required, a fiberscope or endoscope is used, as shown in Figure 3. A camera on the fiberscope or endoscope captures the visualized force information and enables estimation of the force values. In this case, surgeons are provided with quantitative values of the retraction force.

### 3.2. Design Requirements

The design requirements for the proposed silicone retractor are as follows:

1. The retraction force can be visualized.
2. The retraction force can be measured while retraction is being performed.
3. The device can be attached to suction pipes.
4. The device does not include any electric components.
5. The device dimensions should be as follows: a width of less than 30 mm, length of less than 20 mm, and thickness of less than 15 mm.
6. The force can be measured over a range of 0–0.3 N, with a resolution of 0.05 N.

To deal with both the situations described above, a force-visualization-based mechanism is utilized. The main objective is to enhance usability by enabling simultaneous performance of suction, retraction, and retraction-force sensing. The suction function is provided by the suction system, whereas the retraction function is provided by a silicone retractor attached to the suction pipe. Through addition of a retraction-force sensing function to the silicone retractor, the three functions can be provided simultaneously. For ease of setup and usage, a disposable, low-cost silicone retractor is preferred. A disposable retractor simplifies the disinfection and sterilization processes. It is then preferable that the silicone retractor does not include any electrical components. The nominal dimensions of the original silicone retractor are 23 mm (width) × 18 mm (length) × 2 mm (thickness). As a preliminary step, the target size was set to be less than 30 mm (width) × 20 mm (length) × 15 mm (thickness). The required force range and force resolution were established according to experimental results of conventional studies [39,40,41]. Gan et al. [39] reported that more than 70% of neurosurgical tasks were conducted with a force of less than 0.3 N. The force range was then set to 0–0.3 N. A noticeable difference in force (10%) was reported [42]. In the target situations, surgeons need to pay attention to not only the changes in force but also other tasks, such as suction. The force information acquired by the silicone retractor is not used for control purposes; rather, it is used for being displayed to the surgeon. The most important point to be considered is to refrain from applying a very large load, because this can cause damage to tissues. With these objectives in mind, a resolution of 0.05 N was considered in this study.

### 3.3. Principle of Force Sensing

**Figure 4:**
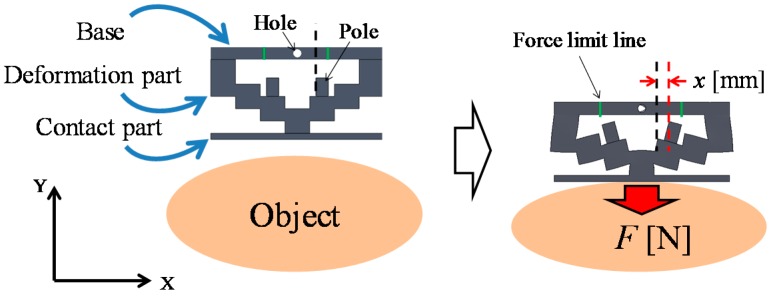
Principle of force sensing.

Figure 4 shows a schematic view of the force-sensing system, which is based on a force visualization mechanism. The X- and Y-axes are defined as shown in Figure 4. Here, *F* (N) denotes the force in the Y-direction and corresponds to the magnitude of the retraction force, and *x* (mm) denotes the distance moved by the tips of the poles (markers) in the X-direction from the initial position. The silicone retractor consists of a base, a deformation part, poles, and a contact part. The base is the reference for deformation and is made of a relatively hard material. The silicone retractor can be attached to the tip of the suction pipe through a hole at the center of the base. The contact part was added to the retractor to enable retraction of a wider area, and it is also made of a relatively hard material. The retraction force is detected at the center of the contact part, thereby permitting derivation of the force on rough or curved surfaces. It should be noted that we are not concerned with force measurement when the tissue is not in contact with the center of the contact part. This is because retraction requires contact with the center of the contact part. The stair-like structure in the deformation part enlarges the deformation due to the retraction force. This stair-like structure deforms with the retraction of tissues. The joint for each stair is thin, and stress concentrations occur at these points. Large deformations are expected owing to these stress concentrations. The pole attached to the stair is a marker that displays the enlarged deformation as force information to the operator. The pole attached to the (second) stair was selected so that the pole can enlarge and display the deformation while tilting and moving without any contact with other areas/walls. The maximum movement occurs at the tip of the pole, which corresponds to the movement distance *x* (mm). The movement distance depends on the magnitude of the retraction force. Thus, in the calibration of the relationship between the distance moved by the pole tip, *x* (mm), and the retraction force *F* (N), the magnitude of the retraction force can be derived from *x* (mm). Therefore, the distance moved by the pole tip can be used to visualize the force information. It should be noted that during contact with tissue at large inclinations, the movement distances *x* (mm) could be different at the left and right poles. In this case, the mean of distances moved by the left and right poles is used to derive the retraction force *F* (N).

### 3.4. Structure of Silicone Retractor with Embedded Force-Sensing System

**Figure 5:**
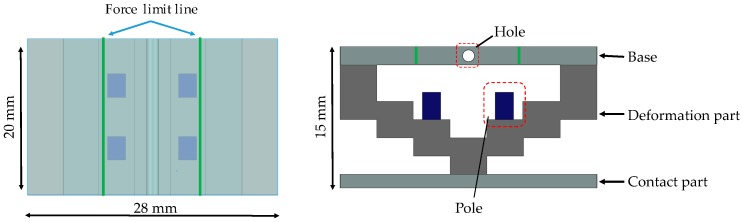
Schematic top and side views of structure of silicone retractor including a force-sensing function.

Figure 5 shows a schematic of the structure of the silicone retractor with an embedded force-sensing function. The retractor consists of a base, a deformation part, a contact part, and poles. All parts are made of silicone, thereby making the retractor disposable, inexpensive, and easy to sterilize or disinfect. The deformation part and poles are made of soft silicone, whereas the base and the contact part are made of hard silicone. The soft silicone and hard silicone were constructed from base materials (KE-1308, Shin-Etsu Chemical Co., Ltd. Tokyo, Japan) and hardeners (CAT1300, Shin-Etsu Chemical Co., Ltd. Tokyo, Japan) procured from Shin-Etsu Silicone Division. The weight ratio of the base material to the hardener was 1:0.06 for the soft silicone and 1:0.1 for the hard silicone. The base was made transparent to enable observation of the movement of the pole from the top. The pole was colored with K-COLOR-BL-70 (Shin-Etsu Chemical Co., Ltd. Tokyo, Japan). Colored lines were drawn on the base so that the operator could see the force limits. The lines were drawn in ink that is harmless to human tissue. If the tip of the pole goes beyond the line of the force limit, the retraction force becomes greater than the allowable maximum (in this case, 0.3 N). By checking whether the pole tip is beyond the line, the operator can keep the retraction force below the allowable maximum and prevent damage to brain tissues. It should be noted that when the distances moved by the left and right poles are significantly different (for example, owing to a large inclination of the silicone retractor), the mean distance ought to be measured. The top view is intended for observation by surgeons, whereas the side view should be captured with a camera. The dimensions of the silicone retractor are 20 mm (length) × 28 mm (width) × 15 mm (thickness). The dimensions of the poles are 3 mm (length) × 2 mm (width) × 3 mm (thickness).

**Figure 6:**
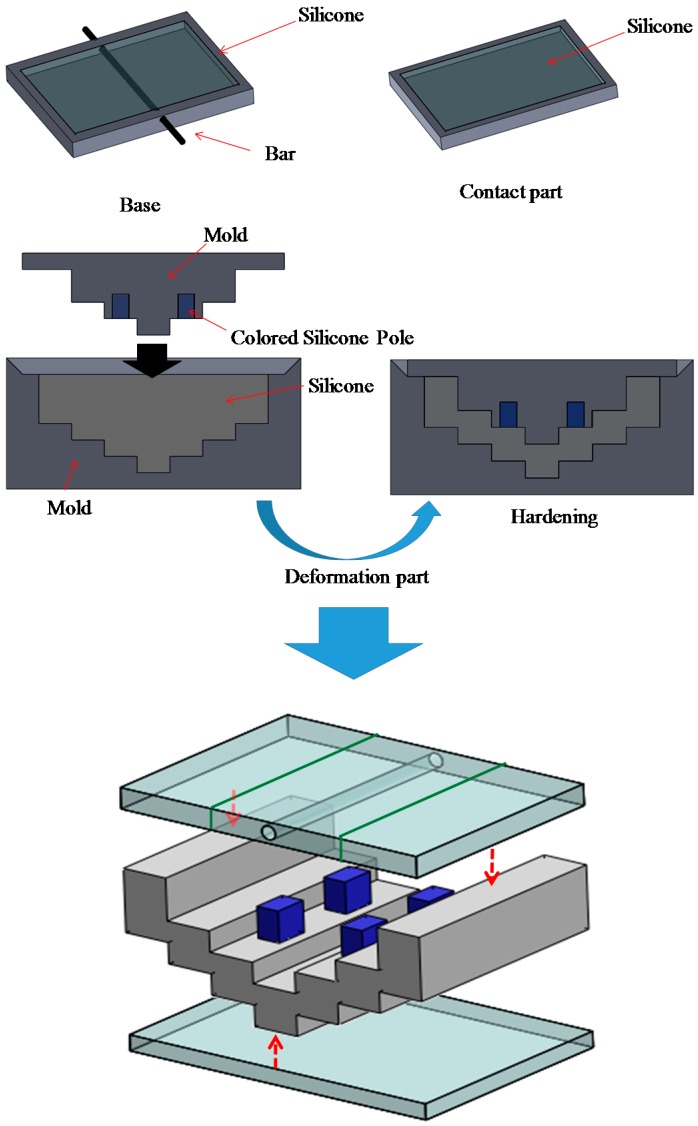
Overview of manufacture and assembly processes of silicone retractor including a force-sensing function.

**Figure 7:**
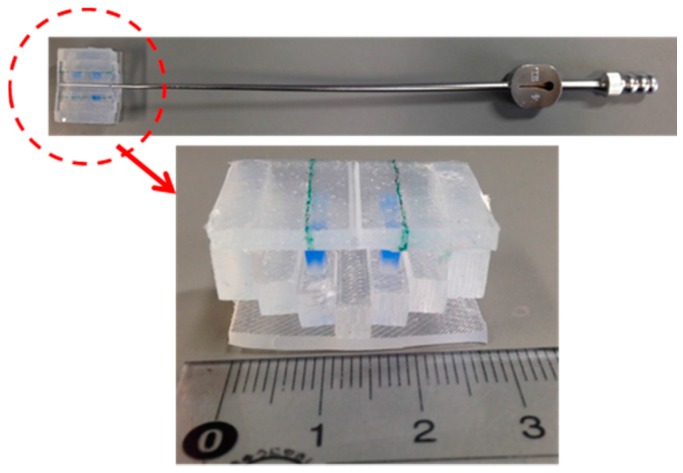
Manufactured silicone retractor including a force-sensing function.

Figure 6 shows an overview of the manufacturing and assembly processes. First, the poles are manufactured using a mold. After the poles have been placed into the holes in the mold for the deformation part, liquid silicone is poured into the molds, and the deformation part is manufactured. The base is manufactured using a mold with a bar. The contact part is also manufactured using a mold. At the time of manufacture of the base, the base and the deformation part can be joined. Similarly, the contact part can be joined to other parts during its manufacture. To simplify the manufacture of the prototype, bonds were used for jointing. The manufactured retractor is shown in Figure 7.

### 3.5. FEM Analysis of Relationship between Retraction Force and Distance Moved by Pole Tip

Finite element method (FEM) analysis was conducted to validate whether the stair-like deformation part can visualize the force, as well as to determine the relationship between the retraction force *F* (N) and the distance *x* (mm) moved by the pole tip in the X-direction (see the coordinates in Figure 4). SolidWorks Simulation (SolidWorks) was used for the FEM analysis. The undersurface of the contact part was fixed, and a load was applied at the hole in the base in the negative Y-direction. The load was changed from 0 N to 0.3 N in increments of 0.05 N. Table 1 lists the material properties of each part. The material properties were set according to the stress—strain diagram derived from experimental results. A linear elastic isotropic model was used for the simulation, and a tetrahedron was selected as the element by taking into account the material properties of the used silicones. (It should be noted here that the viscosities of the used silicones were negligible.) The materials for the base and the contact part were different from that for the deformation part, and accordingly, different values of material properties were used (see Table 1).

**Figure 8:**
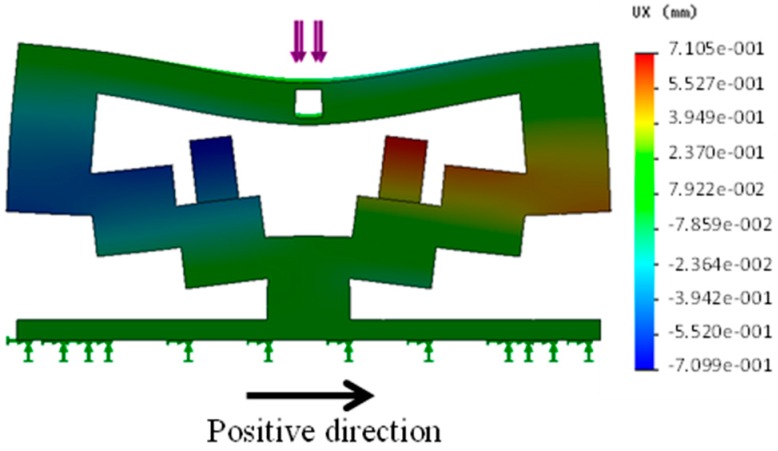
Results of FEM analysis under applied load of 0.1 N.

Figure 8 shows the FEM analysis results for an applied load of 0.1 N. The largest movement distance was obtained at the top-left and top-right points of the right and left poles. Therefore, the force information could be obtained by checking the distances moved by the pole tips. To estimate the force value, the relationship between the load *F*(N) and the distance *x* (mm) moved by the pole tip was calibrated. The distances moved by the pole tip, *x* (mm), for each *F* (N) are plotted in the figure at Section 4.1.3. The exact relationship is derived in the next section.

## 4. Experimental Evaluation

### 4.1. Case of Retracting a Flat and Solid Surface

**Figure 9:**
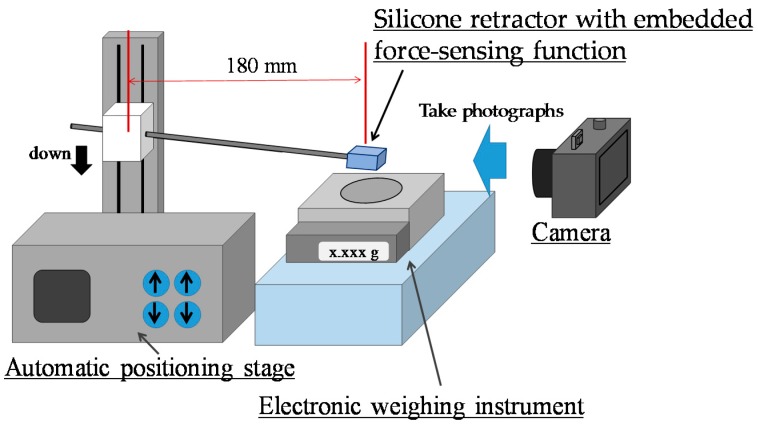
Schematic of experimental setup.

**Figure 10:**
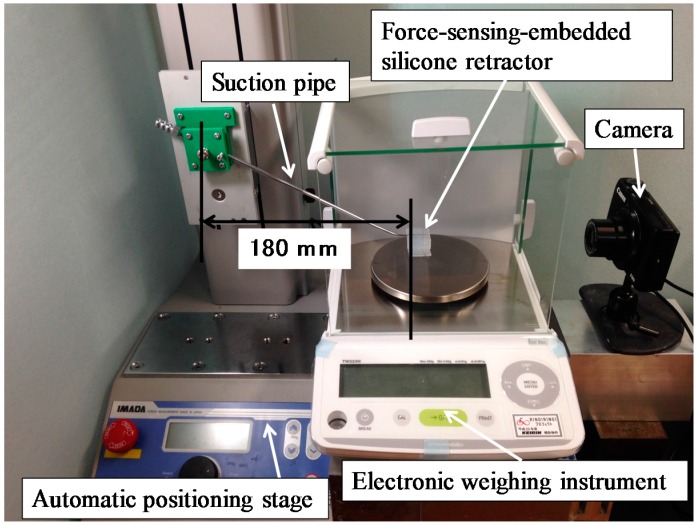
Photograph of experimental setup.

Experiments were conducted to validate the performance of the proposed silicone retractor embedded with a force-sensing function and to determine the relationship between the distance moved by the pole tip (in the X-direction), *x* (mm), and the retraction force *F* (N). Figure 9 shows a schematic of the experimental setup, and Figure 10 shows a photograph of the actual setup. A suction pipe equipped with the developed silicone retractor was attached to an automatic positioning stage (IMADA MX2-500N), which precisely controlled the magnitude of the load by moving the suction pipe in the vertical direction. The silicone retractor was placed on the stage of an electronic weighing instrument (SHIMADZU TW223N) to measure the load applied to the retractor. A digital camera (CANON PSS120BK) was used to capture the movement of the poles in the silicone retractor. Table 2 presents the relevant specifications of the experimental devices.

#### 4.1.1. Procedure

**Figure 11:**
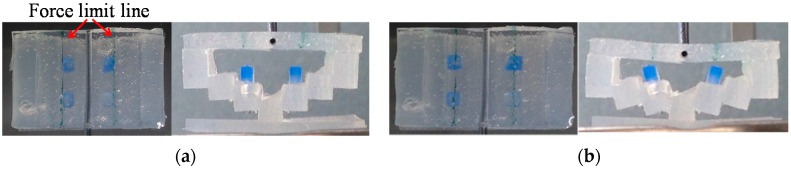
Photographs of silicone retractor at loads of (**a**) 0.00 N and (**b**) 0.2 N.

First, the automatic positioning stage was moved so that the silicone retractor was in contact with the stage of the electronic weighing instrument and the instrument displayed “0.000 g” (0.00 N). The automatic positioning stage was then controlled to increase the magnitude of the load by 0.05 N. After this control, a photograph was taken with the camera to measure the distance moved by the pole tip, *x* mm. This procedure was repeated for loads in the range of 0.00–0.30 N in increments of 0.05 N. The entire procedure was repeated five times. As a result, 35 photographs were obtained in total. Figure 11 shows representative photographs obtained at loads of 0.00 and 0.2 N.

#### 4.1.2. Derivation of Distance Moved by Pole Tip in Silicone Retractor

**Figure 12:**
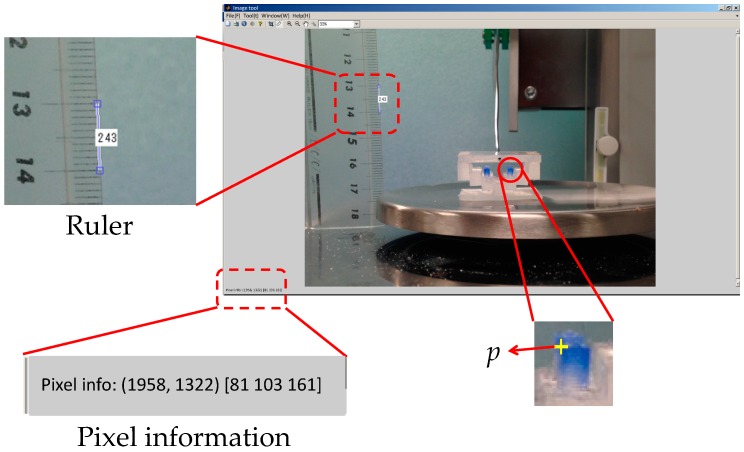
Derivation of distance moved by pole tip by using Imtool.

The image processing toolbox of MATLAB (MathWorks) was used to derive the distance moved by the pole tips. Imtool (MATLAB) was used to obtain the pole positions and information for unit conversion. The motion of the right pole was examined by taking into account the symmetry of the silicone retractor. In Figure 12, p(f) is the position of the top-left point of the right pole and *F* is the applied load. Imtool was used to derive p(f) for every load *F*. For the unit conversion, a ruler was also photographed using the camera, as shown in Figure 12. The pixel value corresponding to *x* of 10 mm was derived, and the units were converted from pixels to millimeters. The distance moved by the pole tip in the X-direction was derived as $\left|p_{x}\left(f\right)-p_{x}\left(0.00\right)\right|$, where $p_{x}\left(f\right)$ denotes the X-coordinate of p(f). We focused on the movement of the X-coordinate because it was significant and easy to detect, as shown in Figure 4. Note that image processing could have been applied to determine the movement, but a manual derivation was instead used to acquire accurate results. 

#### 4.1.3. Relationship between Retraction Force and Distance Moved by Pole Tip

**Figure 13:**
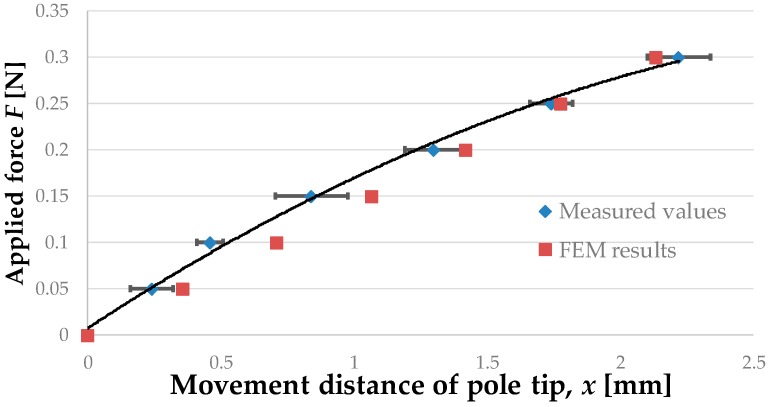
Relationship between retraction force and distance moved by pole tip.

Figure 13 shows the experimental results (blue diamonds) and the values obtained by FEM analysis (red squares). Mean values with error bars expressing the standard deviation are given for the experimental results. The regression curve for the experimental results is

(1) $$F=-0.026x^{2}+0.19x+0.0089$$

It should be noted that we did not consider forces greater than 0.3 N, because these forces are beyond the range of use of the proposed device.

Table 3 presents the properties of the regression curve, i.e., the obtained coefficient of determination and the root mean squared error (RMSE). The calculation was performed using the curve fitting toolbox of MATLAB (MathWorks).

#### 4.1.4. Discussion

The results of the experiments and FEM analysis were found to differ slightly (RMSE of 0.022). This difference may perhaps have been due to the difficulty in setting the material parameters in the FEM analysis to be the same as those in the actual silicone retractors. The shapes of the manufactured silicone retractors were also not identical to those used in the FEM analysis. However, the obtained values were close to each other. Thus, the experimental results were validated by the FEM analysis. The distance moved by the pole tips increased monotonically with the applied load. The regression analysis results (Figure 13 and Table 3) show that a quadratic function can express the relationship very well. Hence, the retraction force can be estimated from the distance moved by the pole tips. The resolution of the force-sensing system was less than 0.05 N, and the force range achieved by the sensor was 0–0.3 N. 

The left-hand-side images in Figure 11a,b show the relation between the position of the blue-colored pole and the line expressing the force limit. This relation allows for rough estimation of the force. Even if there is no camera or fiberscope, the surgeon can estimate the retraction force by using the developed silicone retractor with an embedded force-sensing function.

### 4.2. Case of Retraction of Curved and Soft Surfaces

**Figure 14:**
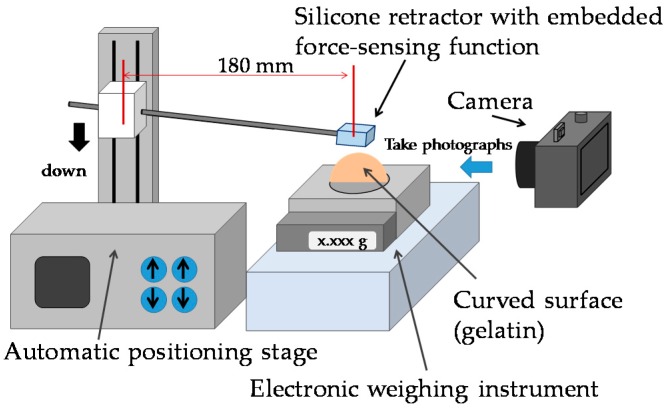
Schematic of experimental setup in the case of retraction of curved and soft surfaces.

**Figure 15:**
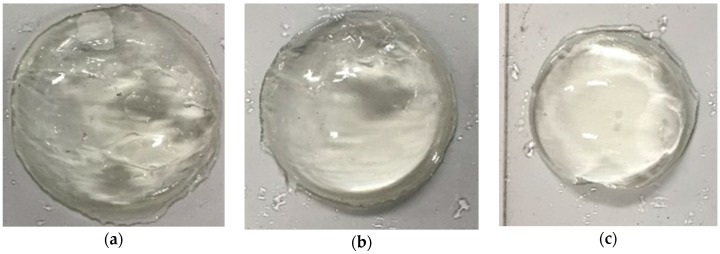
Curved surfaces made of semi-spherical gelatin: (**a**) Large (radius: 3 mm); (**b**) Medium (radius: 2.5 mm); and (**c**) Small (radius: 2 mm).

**Figure 16:**
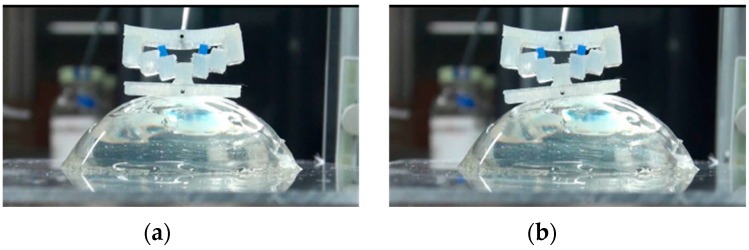
Photograph of case of retracting large-sized gelatin curved surface ((**a**) 0.00 N; (**b**) 0.2 N).

**Figure 17:**
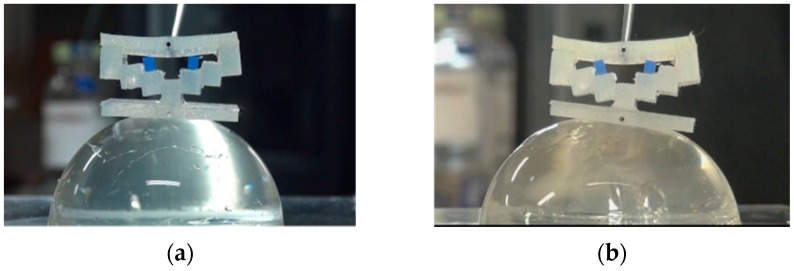
Photograph of case of retracting medium-sized gelatin curved surface ((**a**) 0.00 N; (**b**) 0.2 N).

**Figure 18:**
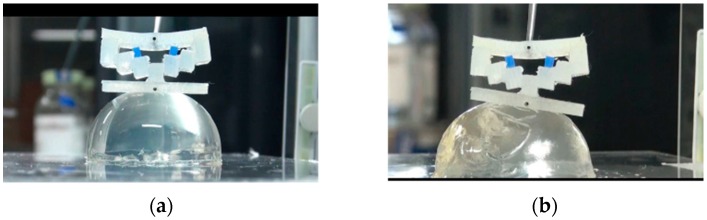
Photograph of case of retracting small-sized gelatin curved surface ((**a**) 0.00 N; (**b**) 0.2 N).

In real situations, the target tissue may be soft and curved. To investigate the performance of the developed force-sensing silicone retractor with such tissues, the relationship between the distance moved by the pole tip (in the X-direction), x mm, and the retraction force f N was identified for the case of curved and soft surfaces. The experimental setup shown in Figure 14 is the same as that employed in the first experiment (Figure 9); however, different target objects were used in these experiments. The target objects were semi-spherical gelatins. Three different sizes (small, medium, and large; see Figure 15) of gelatins were prepared to examine the effects of different curvatures on the performance of the retractor. As can be seen from Figure 15, the surfaces of the gelatins were not perfectly smooth; rather, they were somewhat rough because the gelatins were handmade. Therefore, this evaluation can be regarded as being nearly the same as that for the case of rough surfaces. Figure 16, Figure 17 and Figure 18 show photographs of the gelatins under representative loads of 0.00 and 0.2 N.

#### 4.2.1. Procedure

The experimental procedure was essentially the same as that for the previous experiment. The automatic positioning stage was moved so that the silicone retractor was in contact with the curved surface on the electric weighing instrument while the latter displayed a reading of “0.000 g” (0.00 N). The automatic positioning stage was then controlled to increase the magnitude of the load by 0.05 N. After this control, a photograph was taken with the camera to measure the distance moved by the pole tip, *x* mm. Two retraction positions were examined: one was at the center of the curved surfaces and the other was at a distance of 5 mm from the center. The entire procedure was repeated five times for each case. As a result, 210 photographs were obtained in total. The distance moved by the pole tip on the silicone retractor was derived by averaging the distances moved by the left and right pole tips. The objective of this averaging was to reduce the effect of differences between the left and right pole tips resulting from skewed retraction directions (see Figure 16, Figure 17 and Figure 18).

#### 4.2.2. Relationship between Retraction Force and Distance Moved by Pole Tip

**Figure 19:**
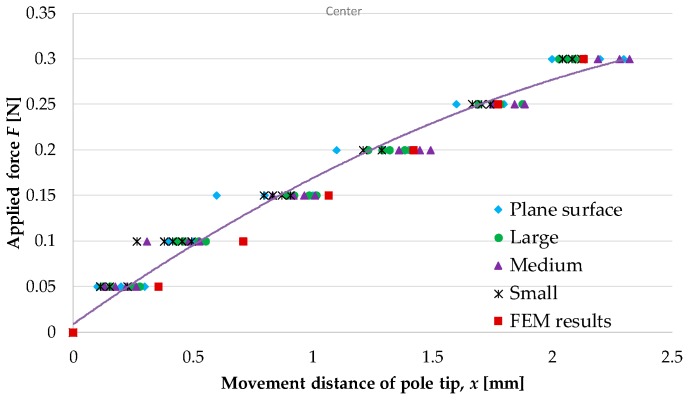
Relationship between retraction force and distance moved by pole tip for the case of retraction at center of curved surfaces; the results shown in Figure 13 are also included here for comparison purposes.

**Figure 20:**
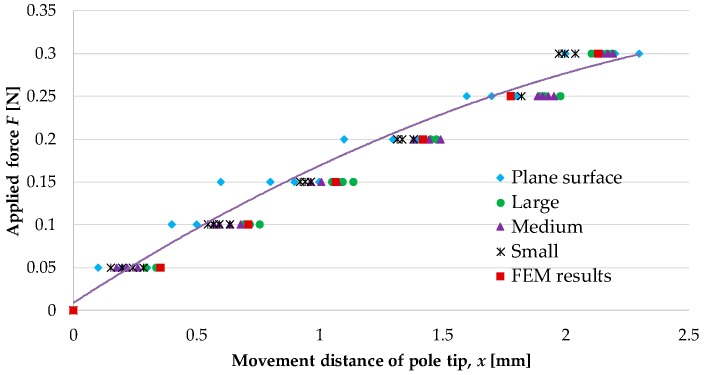
Relationship between retraction force and distance moved by pole tip for the case of retraction at a 5 mm distance from center of curved surfaces, the results shown in Figure 13 are also included here for comparison purposes.

Figure 19 and Figure 20 show the relationship between the retraction force f N and the distance moved by the pole tip, x mm, for the cases of retraction at the center of the curved surfaces and retraction at a distance of 5 mm from the center of the curved surfaces, respectively. Both figures show the measured results as well as the results shown in Figure 13 obtained using the regression curve given in Equation (1). Table 4 presents the RMSE between the measured values and the values derived by the regression curve.

#### 4.2.3. Discussion

The experimental results shown in Figure 19 and Figure 20 are very close to the regression curve, and they reveal that the sensor resolution was less than 0.05 N in all cases. This is also confirmed from the data in Table 2. These results indicate that the effects of curvature and retraction position on the performance of the retractor are relatively weak. It can be seen from Figure 16, Figure 17 and Figure 18 that the direction of retraction in these cases deviated from that in the case depicted in Figure 20. Nonetheless, the effects of the deviation of the retraction direction on the results are again weak. This could be because the operation point was concentrated at the center of the contact part (see Figure 4) or because the sensor has a measurement range of 0–0.3 N. The experimental results thus validated the performance of the developed force-sensing system. 

## 5. Conclusions

This paper has described a silicone retractor with an embedded force-sensing function that can be attached to a suction pipe. Suction pipes and retractors are the most frequently-used medical devices in neurosurgery. Recently, we developed a device that provides a combination of the suction and retraction functions [1]. However, the device was not equipped with a force-sensing function. Estimation of the retraction force allows for safer and more precise operation. This paper presented a device that can provide all three functions—suction, retraction, and retraction-force sensing—simultaneously. This force-sensing system was embedded into the developed silicone retractor, which is attached to the tip of a suction pipe. A force visualization mechanism was utilized for force sensing. The embedded colored pole moves or tilts upon application of a retraction force. The distance moved by the pole tip corresponds to the magnitude of the retraction force, and so, the retraction force can be estimated from the distance moved by the pole tip. This aids surgeons in estimating the retraction force. If a camera or fiberscope is used, the force can be measured with a resolution of 0.05 N up to a maximum value of 0.3 N. The effects of the curvature and retraction positions of targets are negligible, as the operation point of the device is concentrated in a small area. Since the developed retractor is made of silicone, it is disposable, inexpensive, and easy to sterilize or disinfect. The developed silicone retractor is expected to not only reduce the number of times tools need to be switched, but also enable safer and more precise retraction.

We received positive feedback from surgeons about the usability of the force-sensing system. Surgeons often check the retraction point at which the force-sensing system is embedded during surgical operations. Therefore, the force value can be checked easily by using the force-sensing system. Nevertheless, conducting feasibility studies (for example, investigation of how accurately surgeons can check the retraction force during operations) in real situations will be one of our future endeavors.

The limitations of the proposed force-sensing system are as follows. The retraction force cannot be recorded in the absence of an endoscope. If retraction is performed by robots, higher resolution is required for force control. The presently achieved resolution of sensing is not high enough for force control by robots. Furthermore, when a surgeon is approaching a deep area where the retractor cannot be seen directly, the retraction force cannot be measured owing to the occlusion of the force display area. Additionally, in order to enable insertion of the retractor into a deep area, its thickness may need to be made smaller than it is at present.

For practical applications, further miniaturization and evaluation of the proposed force-sensing system in real situations are required. These issues will be considered in future studies.

## Figures and Tables

**Table 1:** Material properties in FEM analysis.

Part	Young’s Modulus MPa	Poisson’s Ratio
Base	0.5	0.3
Deformation part	0.2	0.3
Contact part	0.5	0.3

**Table 2:** **R**elevant specifications of experimental devices.

Device	Camera	Electronic Weighing Instrument	Automatic Positioning Stage
Resolution	2816 × 2112 pixels	0.001 g	0.01 mm
Speed	-	-	10 (mm/min)

**Table 3:** Properties of regression curve.

Dimension of Polynomial Function	Coefficient of Determination, R^2^	RMSE
2	0.98	0.014

**Table 4:** RMSE between measured values and those derived from regression curve (Equation (1)).

Retraction Position	Large	Medium	Small
Center	0.013 N	0.017 N	0.012 N
At a distance of 5 mm from center	0.026 N	0.020 N	0.014 N
